# SncRNA (microRNA & snoRNA) opposite expression pattern found in multiple sclerosis relapse and remission is sex dependent

**DOI:** 10.1038/srep20126

**Published:** 2016-02-01

**Authors:** Maider Muñoz-Culla, Haritz Irizar, Matías Sáenz-Cuesta, Tamara Castillo-Triviño, Iñaki Osorio-Querejeta, Lucía Sepúlveda, Adolfo López de Munain, Javier Olascoaga, David Otaegui

**Affiliations:** 1 Biodonostia Institute, Neuroscience Area, Multiple Sclerosis Group, San Sebastian, Spain; 2 REEM: Spanish network on Multiple Sclerosis (Red española de esclerosis múltiple –REEM-), Casanoves, 143, Recepción Edificio CELLEX, 08028 Barcelona, Spain; 3 Institute for Genomics and Multiscale Biology, Department of Genetics and Genomics Sciences, Icahn School of Medicine at Mount Sinai, New York, NY, USA; 4 Hospital Universitario Donostia, Neurology Department, San Sebastian, Spain; 5 University of the Basque Country (UPV-EHU), Department of Neuroscience, San Sebastian, Spain; 6 CIBERNED: Parque científico de Madrid. C/ Faraday 7 -28049 Madrid, Spain

## Abstract

Multiple sclerosis (MS) is a common inflammatory and degenerative disease that causes neurological disability. It affects young adults and its prevalence is higher in women. The most common form is manifested as a series of acute episodes of neurological disability (relapses) followed by a recovery phase (remission). Recently, non-coding RNAs have emerged as new players in transcriptome regulation, and in turn, they could have a significant role in MS pathogenesis. In this context, our aim was to investigate the involvement of microRNAs and snoRNAs in the relapse-remission dynamics of MS in peripheral blood leucocytes, to shed light on the molecular and regulatory mechanisms that underlie this complex process. With this approach, we found that a subset of small non-coding RNAs (sncRNA) is altered in relapse and remission, revealing unexpected opposite changes that are sex dependent. Furthermore, we found that a relapse-related miRNA signature regulated general metabolism processes in leucocytes, and miRNA altered in remission are involved in the regulation of innate immunity. We observed that sncRNA dysregulation is different in relapse and remission leading to differences in transcriptome regulation, and that this process is sex dependent. In conclusion, relapse and remission have a different molecular background in men and women.

Multiple sclerosis (MS) is a common inflammatory and degenerative disease that causes neurological disability in young adults^1^. It is a complex disease with still unknown etiology but with clear evidence for both environmental and genetic components contributing to its development^2^. As in most autoimmune diseases, the prevalence is higher in women (ratio of 2.6:1)^3^. Furthermore, sex effects have also been observed in other aspects of the disease including severity, clinical course, susceptibility, autoimmune response and gene expression^4^,^5^. In the most common form of MS, called relapsing-remitting MS (RRMS), patients have a series of acute episodes of neurological disability lasting at least 24 hours (what is called a “relapse”) followed by a complete or incomplete recovery phase (remission)^6^. These relapses and the incomplete recovery from symptoms lead to progressive degeneration and increasing neurological disability. Nevertheless, the correlation between the relapse rate and clinical symptoms is inexact in many patients, suggesting that there is silent disease activity. Therefore, it is important to clarify the underlying biological processes related to the disease activity in order to develop tools to identify these processes and design better treatment strategies.

Gene expression studies have helped improve our understanding of the biological mechanisms underlying disease^7^,^8^,^9^. On the other hand, initially, all studies of this type have focused on the protein-coding region, which comprises less than 2% of the genome^10^,^11^. Recently, however, interest has turned to the non-coding part of the genome, as its products have been shown to be functional and to play important roles in both physiology and disease^12^. Transcriptional products of all these non-protein coding sequences form a variety of RNAs, the so-called non-coding RNAs, each one with a different size and function.

The most widely studied type of non-coding RNAs are microRNAs (miRNAs), single-stranded RNAs of approximately 22 nucleotides-long that regulate the expression of their target mRNAs in a post-transcriptional fashion^13^. In the last decade, miRNAs have appeared as a new player in the complex network of gene expression regulation and are known to be involved in several biological processes including development, differentiation and immunity^14^,^15^,^16^. It has been shown that they also take part in cancer, neurodegeneration and autoimmune diseases^17^,^18^,^19^. In MS, several studies in recent years have reported differences in miRNA expression between MS patients and healthy donors in leucocytes, serum, MS brain lesion tissue and cerebrospinal fluid (reviewed in^20^). However, the functional and pathological implications of the dysregulation of these miRNAs are still unknown.

Small non-coding RNAs include other subtypes apart from miRNAs and, among them, the small nucleolar RNAs (snoRNAs) have been the most widely studied. They perform sequence-specific 2′-*O*-methylation and pseudouridylation of ribosomal RNA which takes place in the nucleolus after forming the small nucleolar ribonucleoprotein (snoRNPs) complex^21^. A role for snoRNAs in cancer has been described and they have also been proposed as good candidates for involvement in other diseases^12^.

In this context, we wanted to investigate the involvement of miRNAs and snoRNAs in the relapse-remission dynamics of MS in order to shed light on the molecular and regulatory mechanisms that underlie this complex process. For this purpose, we analyzed genome-wide sncRNA (miRNA and snoRNA) expression in relapse and remission samples obtained from the same MS patients and matched healthy controls. Furthermore, and with the aim of gaining insight into the impact of the disease on sncRNA-mediated gene expression regulation, we strengthened our analysis by integrating previously analyzed large-scale mRNA expression data from the same samples, thereby creating miRNA-target gene interaction networks.

## Results

### Differentially expressed sncRNA

**Figure 1:**
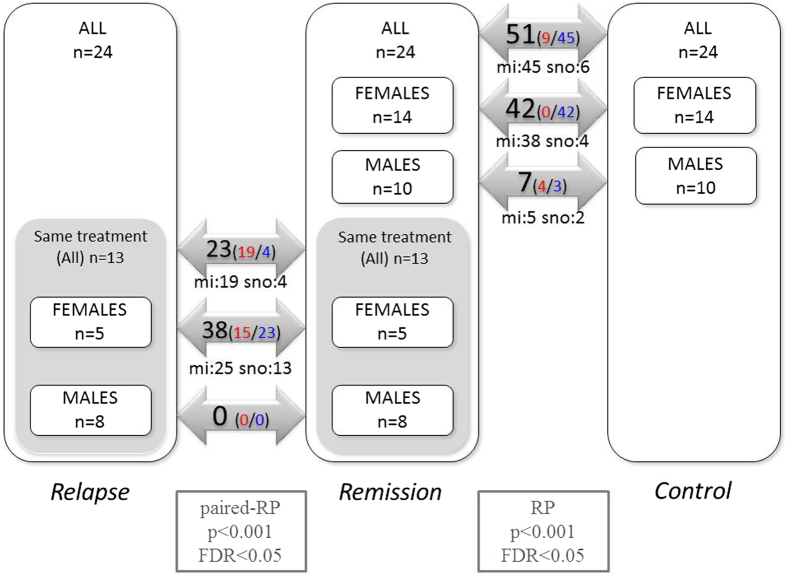
Summary of the results obtained in comparisons using the rank product algorithm. Numbers inside the arrows indicate the number of differentially expressed non-coding RNAs found (false discovery rate < 0.05). Red- and blue-colored numbers are overexpressed and underexpressed ncRNAs, respectively. mi: miRNA; sno: snoRNA.

Out of 1769 probes used, 1113 (62.91%) were expressed in at least one sample. Among these, we identified several differentially expressed small non-coding RNAs (sncRNA) (miRNAs and snoRNAs) in both relapse and remission. When all the samples were included, 23 sncRNAs were found to be differentially expressed in relapse. In the analysis stratified by sex, 38 sncRNAs were differentially expressed in females but none were in males. In addition, in remission, we identified 51 altered sncRNAs overall (all samples), 42 and 7 in female and male samples, respectively (false discovery rate < 0.05) (Fig. 1). A complete list of the differentially expressed sncRNAs is provided in Supplementary Tables 1 and 2.

These results show that alterations in sncRNA expression related to the disease are different in males and females. First, the number of differentially expressed probe sets is higher in females in both comparisons. Second, the distribution of the number of over- and underexpressed sncRNAs in remission differs dramatically between sexes, all of them being underexpressed in females, whereas in males, the distribution is balanced. When considering both males and females in this analysis, a similar trend can be observed, with 45 sncRNAs being underexpressed and 9 overexpressed.

**Figure 2:**
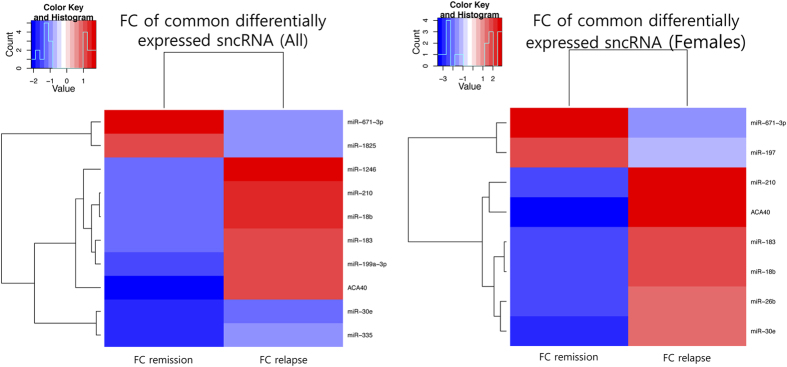
The mirror pattern observed in the sncRNAs altered both in relapse and remission when including all the samples. (**A**) or only female samples (**B**). Fold changes (FCs) are calculated taking the control group as a reference both in relapse and in remission.

After characterizing the transcriptional signature of the sncRNAs in each phase of the disease, we wanted to assess whether any sncRNAs were dysregulated in both relapse and remission, under the hypothesis that such sncRNAs, being dysregulated in both phases of the disease, might play important roles in the biology of the disease. For this purpose, we explored the overlap between the altered sncRNA lists related to relapse and to remission. This analysis identified 10 differentially expressed sncRNAs common between relapse and remission in all the samples, and 8 sncRNAs when including only females. Among these common sncRNAs, 80% in all the samples and 100% in females were dysregulated in opposite directions in relapse and remission comparing the groups to healthy controls (All: p = 0.058; Females: p = 0.004) (Fig. 2). We have called this opposite pattern of sncRNA expression, the “*mirror pattern*”.

Then, by qPCR, we analyzed the expression of the 10 miRNAs that showed the highest fold change in female samples in the microarray experiment. The Pearson’s correlation analysis showed a significant correlation (p < 0.05) between two platforms in 70% of the analyzed miRNAs (data not shown). Seven out of the eight sncRNAs that were altered in relapse in female samples passed the validation by qPCR (Supplementary Figure 1).

### Biological effect of miRNA dysregulation

In order to assess which biological processes could be affected by miRNA dysregulation, we created miRNA-mRNA interaction networks for females in relapse, females in remission and males in remission. As described in the Methods section, these networks are built based on miRNA-target prediction algorithms and mRNA gene expression data obtained from the same samples as those used for miRNA expression analysis^5^.

**Figure 3:**
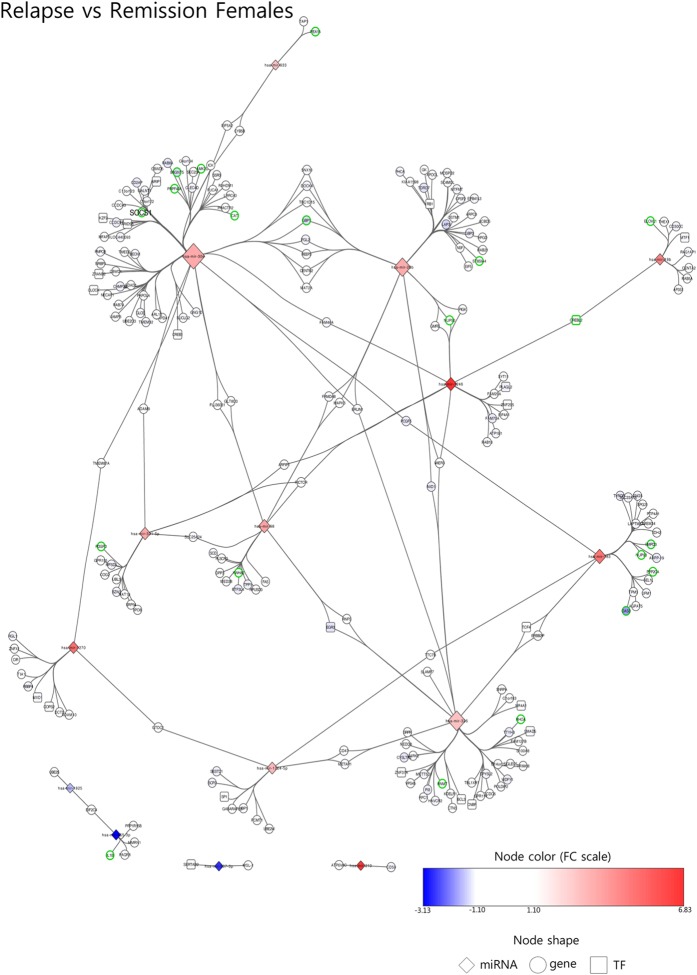
Female relapse network. Built with miRNA altered in relapse vs remission in female samples and their target mRNA as predicted by miRDIP. Node colors show fold change according to the color scale bar. Green-border nodes indicate genes associated with enriched gene ontology terms. Larger node labels indicate genes which have been associated with MS in genome-wide association studies. Node size reflects connectivity (the larger, the more connected). TF: transcription factor. Gene: non-transcription factor gene.

The network created with the miRNAs differentially expressed during relapse in females and their target genes is composed of 232 nodes and 257 edges (representing the possible interactions between miRNAs and mRNAs) (Fig. 3). Among these 232 nodes, there are 15 miRNAs, 22 transcription factors (TFs) and 195 non-TF protein-coding genes. The gene ontology analysis revealed seven biological processes enriched in this network: *regulation of glucose transport, carbohydrate transport, membrane lipid biosynthetic process, membrane lipid metabolic process, regulation of cell division, interferon-gamma (IFNγ)-mediated signaling pathway* and *negative regulation of phosphate metabolic process.*

**Figure 4:**
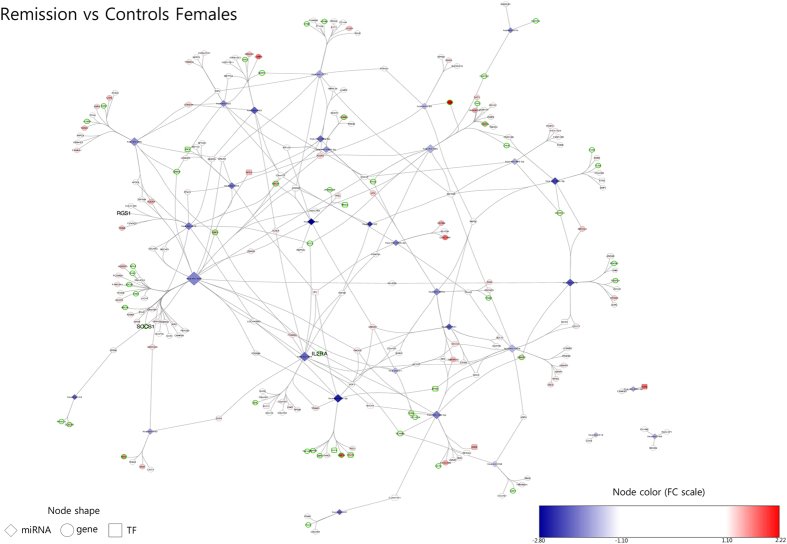
Female remission network. Built with miRNA altered in remission vs controls in female samples and their target miRNA as predicted by miRDIP. Node colors show fold change according to the color scale bar. Green-border nodes indicate genes associated with enriched gene ontology terms. Larger node labels indicate genes which have been associated with MS in genome-wide association studies. Node size reflects connectivity (the larger, the more connected). TF: transcription factor. Gene: non-transcription factor gene.

The network for females in remission is made up of 256 nodes (32 miRNAs, 19 transcription factors and 205 non-TF protein-coding genes) and 340 edges (Fig. 4). These genes show enrichment for 24 biological processes according to the gene ontology analysis. Interestingly, most of these terms are functionally related and can be included in three groups: *innate immune response, regulation of innate immune response* and *homeostasis of number of cells.* On the other hand, the network for males in remission is a low complexity network composed of 25 nodes and 21 edges across four components (Supplementary Figure 2). According to gene ontology analysis, no biological processes are enriched among the genes in this network.

### Expression of miRNA in cultured peripheral blood mononuclear cells

To validate some of our conclusions in an *in vitro* model, miRNA expression was analyzed in PHA-stimulated peripheral blood mononuclear cells (PBMCs) vs non-stimulated PBMCs from a female MS patient and a female healthy donor. Hypothesizing that activated PBMCs could somehow reproduce the gene expression alterations occurring in relapse, we compared results from the two experiments.

First, we tested the activation state of lymphocytes by flow cytometry. We observed that after 72 h, 87.06% of PHA-stimulated cells were positive for CD25 (late-activation marker) and 11.77% were positive for CD69 (early-activation marker) in the MS sample vs 80.03% and 24.48% in healthy controls. On the contrary and as expected, no activation of cells was observed in non-stimulated lymphocytes, in either the MS or the healthy control samples (2.31% CD69^+^ and 2.24% CD25^+^ in MS; and 6.04% CD69^+^ and 6.97% CD25^+^ in healthy control samples).

Microarray expression analysis of the activated MS PBMC population showed altered expression of 413 sncRNAs (201 overexpressed and 212 underexpressed) compared to that in non-activated PBMCs, whereas in the HC sample, 367 dysregulated sncRNAs were found (166 overexpressed and 201 underexpressed). Comparing non-activated PBMC expression from the MS patient with non-activated PBMCs from the healthy control, 68 dysregulated sncRNAs were identified (40 overexpressed and 28 underexpressed). (These lists will be provided under request).

To assess whether the sncRNAs differentially expressed in relapse and remission were also altered in cultured PBMCs, the list of sncRNAs identified as differentially expressed in relapsing patients was compared to that obtained in the stimulated PBMCs. With this analysis, we found that SNORD68 was underexpressed and miR-18b, miR-210 and miR-98 were overexpressed in relapse and PHA-activated MS PBMCs. Another six miRNAs were also altered in both experiments but in the opposite direction. On the other hand, both in remission and in non-stimulated MS PBMCs miR-199a-3p, miR-199b-3p, miR-27b and miR-494 were underexpressed compared to in healthy controls. The expression levels of seven further miRNAs were altered in the opposite direction in blood and cell culture experiments.

## Discussion

Our results reveal a distinct sncRNA differential expression pattern for each phase of the MS. To our knowledge, this is the first time that such differences between relapse and remission have been reported. These results are in accordance with previous gene expression studies in which a relapse-related gene signature has been found^5^,^22^,^23^,^24^ suggesting that the differences found in the transcriptome are, at least partially, regulated by sncRNAs.

Further, the set of sncRNAs that show differential expression in blood leucocytes from MS patients is not the same in males and females. In remission, the pattern of altered sncRNAs in females is markedly different from that in males, and the difference is even more evident in relapse, where 25 sncRNAs are altered in females, but none are found to be dysregulated in males. Moreover, only four out of seven sncRNAs altered in males in remission are coincident with those altered in females in remission. This observation agrees with data of the gene expression study performed by our group^5^ and it is also in line with other studies that describe sex-specific alterations in the transcriptome and the miRNome^25^,^26^,^27^,^28^. These data support the idea that the disease pathophysiology and the process of relapse are molecularly different in males and females.

Several authors have observed that the expression of miRNA is regulated by sexual hormones^29^,^30^. Interestingly, some of the miRNAs found to be underexpressed in our female samples in remission, such as miR-21, miR-181a, miR-26b and miR-27b, have been shown to be repressed by estradiol treatment in breast cancer cell lines^31^. Moreover, it has also been reported that the miR-17-92 cluster, which is altered in our relapse samples, is induced by estrogen receptor alpha^32^. These studies hint at the idea that sex hormones might affect miRNA expression in MS patients. Furthermore, it has also been described that disease modifying therapies modify the expression of aberrantly expressed miRNA^33^,^34^,^35^,^36^ and therefore, more research should be performed to directly address these issues.

The influence of sex on gene expression and its regulation may be among the mechanisms that underlie the sex effects observed in MS at several levels from disease susceptibility to clinical presentation and progression^37^,^38^,^39^. Furthermore, it has been observed that the prevalence of most autoimmune diseases is higher in females, suggesting a role of a general immune mechanism that would cause an increased risk for autoimmunity^1^. Therefore, all these studies suggest that sex should be taken into account in treating MS patients, given differences between males and females in the molecular pathological mechanisms.

**Figure 5:**
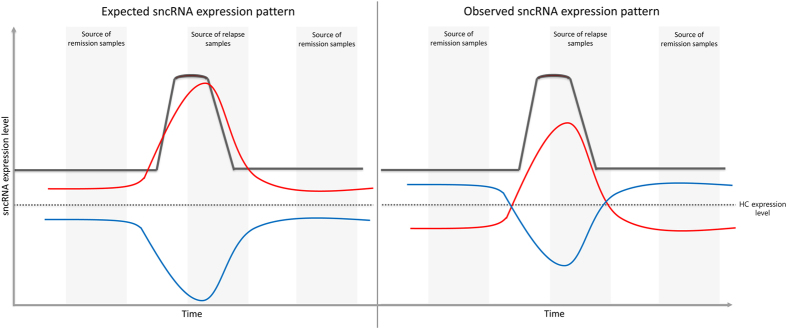
A model to explain the inverted sncRNA expression observed in relapse and remission (the “*mirror pattern”*). The expected pattern for an upregulated (red) or downregulated (blue) sncRNA is shown in the left panel. For a given sncRNA, if it is upregulated in patients in remission compared to in healthy controls, we expected to find it more upregulated in relapse (red line). In the left panel, a model is depicted to explain the observed sncRNA expression pattern. An sncRNA which is underexpressed during remission is found to be overexpressed during relapse compared to healthy controls (red line), and vice versa, an sncRNA which is overexpressed during remission appears to be underexpressed during relapse, compared to healthy controls (blue line).

Since an exacerbation event is a worsening of clinical symptoms, what we expected for a given dysregulated sncRNA in relapse was to find it altered in the same direction as in remission. For instance, if a given sncRNA was overexpressed in remission, we expected to find it more overexpressed in relapse. Yet, surprisingly, we found that sncRNAs that are altered in both phases are dysregulated in opposite directions (i.e., an sncRNA overexpressed in remission is underexpressed in relapse when comparing both to healthy controls, and vice versa). We called this unexpected inverted expression pattern the *mirror pattern.* Interestingly, this *mirror pattern* was also observed in the transcriptomic analysis performed on these patients^5^, and this suggests that both the transcriptome and its regulation via sncRNAs are dynamic and, therefore, can vary during disease progression. In line with this, we propose a model to explain this pattern, in which the expression level of a given sncRNA found to be underexpressed in the pathological state (i.e., during the remission phase) increases during relapse, until the relapse resolves, when the sncRNA expression returns to its initial level (Fig. 5). For an sncRNA that is underexpressed during the remission phase, the reverse would occur.

Aiming to understand the consequences of the dysregulation of miRNAs, we searched for their predicted target genes and considered these together with fold-change data obtained in the gene expression microarray experiment performed on these samples^5^. With this analysis, we combined prediction and experimental data to find relevant miRNA-mRNA interactions, these being visualized as networks. This approach has provided insight into the importance of each of the miRNAs in the regulation of the disease-related genes, highlighting the differences in the networks between relapse and remission. The gene enrichment analysis revealed seven biological processes in the relapse network, pointing to increased cell activity (regulation of glucose transport, carbohydrate transport, membrane lipid biosynthetic process, regulation of cell division and negative regulation of phosphate metabolic process). These observations agree with other research, in which terms related to cell cycle, molecule transport, cell signaling and carbohydrate metabolism were found to be enriched among differentially expressed genes in relapse^24^.

Interestingly, the interferon-gamma (IFNγ)-mediated signaling pathway is also enriched in the relapse network, a pathway involved in CNS inflammation during autoimmune attack^40^. One of the genes participating in this pathway is suppressor of cytokine signaling 1 (SOCS1), an inhibitor of cytokine signaling which has been associated with MS susceptibility^41^. Our results show that SOCS1 is underexpressed in relapse which is consistent with a pro-inflammatory response. The expression, activation and stability of SOCS1 are tightly controlled^42^ and, recently, it has been described that miRNAs are involved in SOCS1 regulation, either directly or indirectly through vitamin D^43^,^44^. Our data suggest that SOCS1 might be regulated by two miRNAs of the same family (miR-30e and miR-30a) given that they have opposite expression in relapse and remission. Furthermore, miR-30e interacts with another two genes involved in IFNγ signaling pathways in the relapse network suggesting that miR-30e induction during relapse might help trigger a pro-inflammatory response targeting several genes involved in IFNγ signaling.

Regarding genes targeted by dysregulated miRNAs in remission, they participate in several biological processes related to the innate immune response and the homeostasis of several types of lymphocytes. Though MS pathogenesis has been classically described as being mediated by adaptive immunity, there is evidence supporting the view that a crucial role is also played by innate immunity^45^,^46^. In fact, a gene expression study conducted in different cell types from relapsing-remitting, secondary-progressive and primary-progressive MS patients revealed an involvement of genes related to innate immunity^47^ and so did our transcriptomic analysis of these patients, neutrophils being found to play an important role^5^.

Interestingly, snoRNAs are also differentially expressed both during relapse and during remission. In fact, one of the RNAs showing the highest dysregulation in females during relapse (fold change of 9.21) is ACA40, which is one of the transcripts also showing the *mirror pattern.* Moreover, this snoRNA has been described to have a central role in the regulation of gene expression in MS according to co-expression networks and it has been suggested as a potential therapeutic target in MS^48^. The results from the *in vitro* experiment validated the differential expression of several sncRNAs, which might help in deciphering the role of altered miRNAs in MS pathophysiology. With this comparison, SNORD68 emerges as a candidate for further studies, given that it is underexpressed after PBMC activation in culture and during relapse in patients. On the other hand, miR-18b, miR-210 and miR-98 are overexpressed in this condition, suggesting that they might play a role in PBMC activation. Interestingly, miR-18b was found to be a candidate biomarker for relapse in previous work by our group^49^.

To conclude, our data highlight the influence of sex on how the immune system responds, and therefore, its importance in MS and other autoimmune diseases. They also show the relevance of the regulation that miRNAs exert on the transcriptome in different stages of the disease. What is more, our data reveal an unexpected dysregulation of a subset of sncRNAs, which are altered in opposite directions in relapse and remission samples compared to healthy controls. Lastly, the importance of other types of sncRNAs such as snoRNAs has been underlined and this points to new avenues of research that may shed some light on MS pathophysiology.

## Methods

### Blood sample collection

Whole blood (10 ml) was collected from 24 patients with RRMS and 24 healthy donors in the Department of Neurology at Donostia University Hospital. In order to analyze the phenomena occurring during relapse, we collected two samples from each MS patient: one during a relapse and another during a remission. A relapse was defined as an episode of new neurological symptoms of at least 24-h duration, not associated with fever or infection^50^. Relapse blood samples were collected before giving any corticosteroid treatment. Healthy donors were matched for age and sex. The main clinical and demographical characteristics of the patients are summarized in Supplementary Table 3. Samples from all donors were collected after receiving written informed consent. The study was approved by the hospital’s ethics committee and samples have been processed and stored at the Basque Biobank (www.biobancovasco.org). The methods were carried out in accordance with the approved guidelines.

### PBMC cultures

Peripheral blood (16 ml) was collected from a healthy donor and an MS patient (Supplementary Table 6) in sodium heparin tubes (Vacutainer, Becton Dickinson). PBMCs were isolated using the Ficoll-Hypaque density gradient method within 2 h of sampling. Cells were frozen until used in RPMI medium 1640 with L-Glutamine (Gibco, Thermo Fisher) supplemented with 10% fetal bovine serum, 10,000 U/ml penicillin, 10,000 μg/ml streptomycin and 10% DMSO. After thawing, cells were resuspended in the same RPMI medium and cultured at a density of 10^6 ^cells/ml in 48-well plates (0.2 ml per well). Under the hypothesis that an activated PBMC population might resemble the state of relapse in patients, we induced the activation of cells stimulating them with phytohemagglutinin (PHA) (Gibco, Thermo Fisher) at 0.5%. Cells were incubated for 72 h at 37 °C and 5% CO_2_. With this protocol, we obtained both PHA-stimulated and non-stimulated PBMCs from an MS patient and a healthy control (PHA-MS, nonPHA-MS, PHA-CNTRL and nonPHA-CNTRL).

### RNA isolation

Total RNA was isolated from peripheral blood leucocytes with the LeukoLOCK kit (Ambion) using the alternative protocol to capture small RNAs. RNA from cultured PBMCs was isolated using the miRNeasy Mini Kit (Qiagen) following the manufacturer’s instructions. RNA concentration was measured using a NanoDrop ND-1000 spectrophotometer and RNA integrity was assessed using a bioanalyzer with the RNA 6000 Nano Assay Protocol (Agilent Technologies). We only included samples with an RNA integrity number higher than 6.

### Microarray hybridization

Total RNA (500 ng) was labeled using the FlashTag Biotin labelling kit (Genisphere) and hybridized to the GeneChip miRNA 1.0 Array (Affymetrix), which covers 847 and 922 human miRNAs and snoRNAs, respectively, following the manufacturer’s instructions. For cultured PBMCs, RNA (300 ng) was labeled using the same kit and hybridized to the GeneChip miRNA 4.0 Array (Affymetrix). This array covers 2578, 2025 and 1996 human mature miRNAs, pre-miRNAs and snoRNAs, respectively.

Briefly, RNA molecules were polyadenylated and, in a subsequent ligation step, a biotin-labeled DNA molecule was attached. Finally, labeled RNA was hybridized to the array, washed and stained in a GeneChip Fluidics Station 450 and scanned in a GeneChip Scanner 7G (Affymetrix).

### Microarray data analysis

#### Blood leucocyte miRNA analysis

We first performed raw data analysis, including a detection step (a probe set is detected above background with an associated p-value) resulting in a true/false call and a quantile normalization step using the miRNA QC Tool software (Affymetrix). In a subsequent filtering step, all non-human probe sets and those that were not detected in any of the arrays were removed, resulting in a final list of 1113 probe sets for downstream analysis. For the differential expression analysis, a class comparison was performed using the MultiExperiment Viewer v4.8.1 software and applying a rank product algorithm with a p-value cutoff of 0.001 and a false discovery rate of less than 0.05. Due to differences between males and females in prevalence and clinical course of the disease, we stratified each group by sex, but we also conducted analysis taking into account all the samples. Therefore, we had three groups for each disease status: all participants, females and males. For comparing relapse and remission, we only considered patients who were receiving the same treatment in both phases of the disease. As a result, we had two subsets: 13 patients receiving the same treatment during relapse and remission (in this case, paired analysis was carried out) and 11 patients receiving different treatments in the two phases. For comparing patients in remission with controls, all 24 patients were included (Fig. 1).

#### Cultured PBMC miRNA analysis

Raw data normalization was carried out in Expression Console software (Affymetrix) using the Robust Multiarray Average algorithm and detection above background was also applied to determine whether probes were detected or not in each array. We selected those expressed in all the arrays. Then, the fold change was calculated comparing PHA-MS vs nonPHA-MS, PHA-CNTRL vs nonPHA-CNTRL and nonPHA-MS vs nonPHA-HC and probe sets with a fold change > |2| were selected. Finally, we compared the resulting lists of altered sncRNA with the ones obtained for the fresh blood leucocyte population.

### miRNA target analysis

For miRNA target prediction, we used the web tool microRNA Data Integration Portal (mirDIP), a database including as many as 12 miRNA prediction algorithms (http://ophid.utoronto.ca/mirDIP/)^51^. Two filters were used: first, a minimum number of 3 databases predicting a given interaction; and second, a minimum average standard score of 40. The standard score given by miRDIP is between 0 and 100 and it is used to evaluate the robustness of the predicted target. The value is a standardization of the scores that each prediction algorithm gives individually. Then, among the predicted targets that passed the two filtering steps, the genes that are expressed in blood were selected, as miRNA regulation will only occur if the target gene is expressed in the same tissue. For this purpose, we used data from another experiment performed by our group, in which the same samples were analyzed using HuGene microarrays^5^. Finally, we only took into account genes that had the opposite fold change to that of their predicted regulator miRNAs, given that there is a general consensus in the literature that miRNAs mainly regulate their target genes by decreasing their expression. After all these steps, we created miRNA-mRNA interaction networks based on both prediction algorithms and biological information recovered from our own samples and visualized them using Cytoscape v3.0.0^52^. As additional information, we highlighted the genes which had been associated with MS in the genome-wide association study published in 2011^53^.

In order to identify biological processes affected by miRNA dysregulation, we carried out a gene ontology enrichment analysis using ClueGO v2.0.2.^54^, a Cytoscape plug-in. Statistical significance was set at a Benjamini-Hochberg corrected p-value of below 0.05.

### qPCR validation

The validation of a selection of differentially expressed miRNAs was performed in the same samples as those studied in the microarray analysis (technical validation) using TaqMan miRNA assays. For validation, we selected 11 miRNAs, namely, those showing the highest fold changes in female samples in the microarray experiment: miR-1246, miR-183, miR-210, miR-671-3p, miR-1270, let-7d*, miR-98, miR-127-3p, miR-382, miR-487b and miR-455-5p. RNA (100 ng) was reverse transcribed using the TaqMan microRNA Reverse Transcription kit (Life Technologies) according to the manufacturer’s protocol. Then, the cDNA (15 ng) was amplified in triplicates in a 7900HT thermal cycler, following the manufacturer’s instructions. RNU48 and U6 were used as endogenous controls given that they are the most widely used and they showed the smallest variance among the three candidate endogenous controls tested (RNU48, RNU44 and U6)(data not shown). The expression level was calculated using the 2^−DDCT^ method^55^ in two groups of miRNAs. On the one hand, we analyzed the miRNAs altered in relapse vs remission in the microarray experiment and, on the other hand, the miRNAs that were differentially expressed in remission vs control. As in the microarray experiment, each analysis was performed in parallel in three groups of samples (*all, females* and *males*).

Furthermore, to assess the concordance between microarray and qPCR data, a correlation analysis was performed by calculating Pearson’s R. To compare the expression of miRNAs between groups a t-test or a Wilcoxon test were used (paired in relapse vs remission) depending on whether or not data followed normal distribution, this being assessed using the Shapiro-Wilk test. Analyses were performed in Excel and R 2.15.0 in RStudio v0.96.330.

### Flow cytometry

After 72 h of incubation, cultured PBMCs were harvested, washed with PBS and incubated with antibodies for 20 minutes at room temperature. Afterwards, cells were washed with PBS and analyzed in a Guava EasyCyte 8HT flow cytometer (Millipore) using the InCyte software v2.2.2. Cell viability was assessed with 7-aminoactinomycin D (7-AAD) (Molecular Probes) and APC-conjugated anti-human CD45 (Becton Dickinson) was used to detect the leucocyte population. Early and late lymphocyte activation was determined measuring the expression of CD69 in cell membrane (PE-Cy^TM^7-conjugated anti-human CD69) and CD25 (PE-conjugated anti-human CD25), respectively (BD Pharmingen^TM^), gated on 7-AAD^−^/CD45^+^ cells.

## Additional Information

**How to cite this article**: Maider, M.-C. *et al.* SncRNA (microRNA & snoRNA) opposite expression pattern found in multiple sclerosis relapse and remission is sex dependent. *Sci. Rep.* **6**, 20126; doi: 10.1038/srep20126 (2016).

## Supplementary Material

Supplementary Information
