# Supplementary material for: SncRNA (microRNA & snoRNA) opposite expression pattern found in multiple sclerosis relapse and remission is sex dependent
Source: Sci Rep. 2016 Feb 1;6:20126. doi: 10.1038/srep20126 (PMC4735588; doi:10.1038/srep20126)
Supplement: Supplementary Information [file srep20126-s1.pdf]

Supplementary information

SncRNA (microRNA & snoRNA) opposite expression pattern found in multiple sclerosis relapse and remission is sex dependent

Muñoz-Culla Maider[1-2], Irizar Haritz[1-2-3], Sáenz-Cuesta Matías [1-2], Castillo-Triviño Tamara [1-2-4], Osorio-Querejeta Iñaki[1-2], Sepúlveda Lucía[1-2], López de Munain Adolfo [1-4-5-6], Olascoaga Javier [1-2-4], Otaegui David [1-2]

[1] Biodonostia Institute, Neuroscience Area, Multiple Sclerosis Group, San Sebastian, Spain

[2] Spanish network on Multiple Sclerosis (Red española de esclerosis múltiple –REEM-)

[3] Institute for Genomics and Multiscale Biology, Department of Genetics and Genomics Sciences, Icahn School of Medicine at Mount Sinai, New York, NY, USA.

[4] Hospital Universitario Donostia, Neurology Department, San Sebastian, Spain.

[4] University of the Basque Country (UPV-EHU), Department of Neuroscience, San Sebastian, Spain.

[6] Centro de Investigación Biomédica en red Enfermedades Neurodegenerativas (CIBERNED

SUPPLEMENTARY FIGURE LEGENDS

Supplementary figure 1. Relative expression of miRNA selected for technical validation. Results of qPCR experiments are shown for seven miRNA that were differentially expressed in relapse vs remission (top panel) and seven miRNA that were altered in remission vs control (bottom panel). *$p<0.05$. **$p<0.01$

Supplementary figure 2. Male remission network. Built with miRNA altered in remission vs controls in male samples and their target miRNA as predicted by miRDIP. Node colors show fold change according to the color scale bar. TF: transcription factor. Gene: non-transcription factor gene.

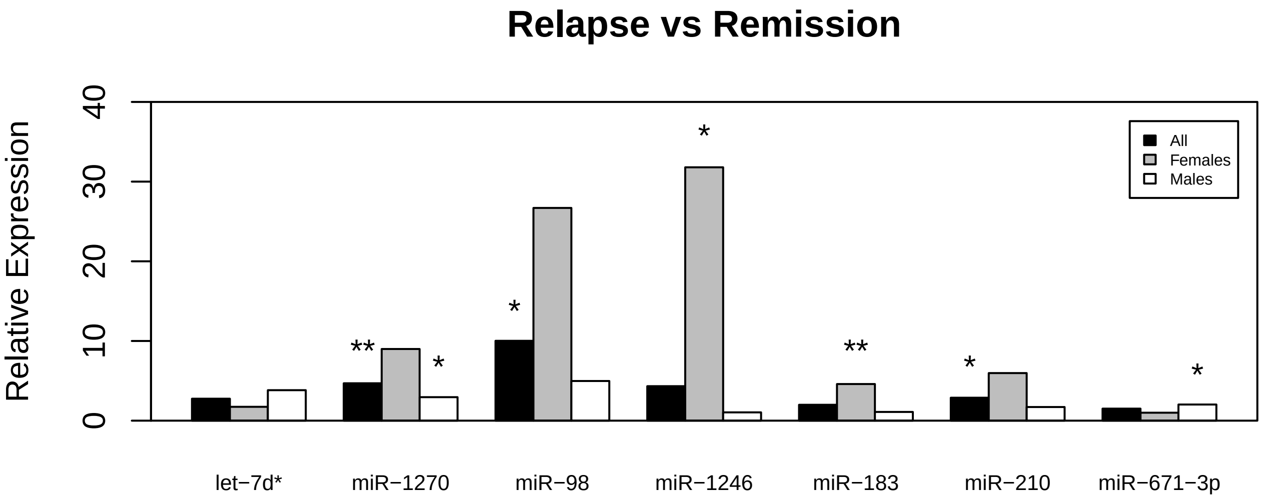
Relapse vs Remission
Relative Expression
0
10
20
30
40
All
Females
Males
let−7d*
miR−1270
miR−98
miR−1246
miR−183
miR−210
miR−671−3p

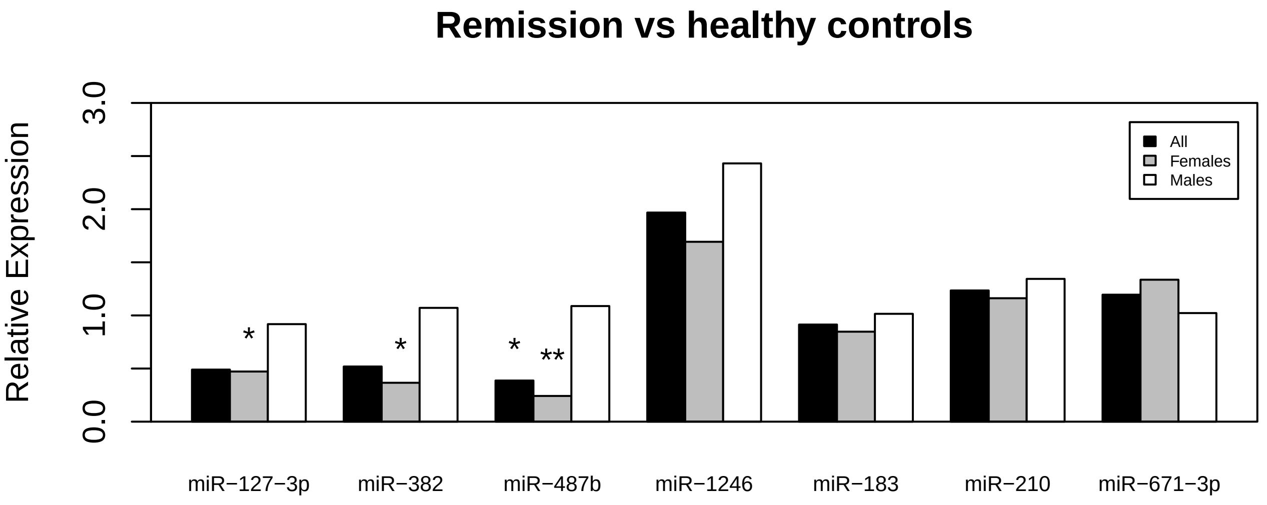
Remission vs healthy controls
Relative Expression
0.0
1.0
2.0
3.0
All
Females
Males
miR−127−3p
miR−382
miR−487b
miR−1246
miR−183
miR−210
miR−671−3p

Remission vs Controls Males

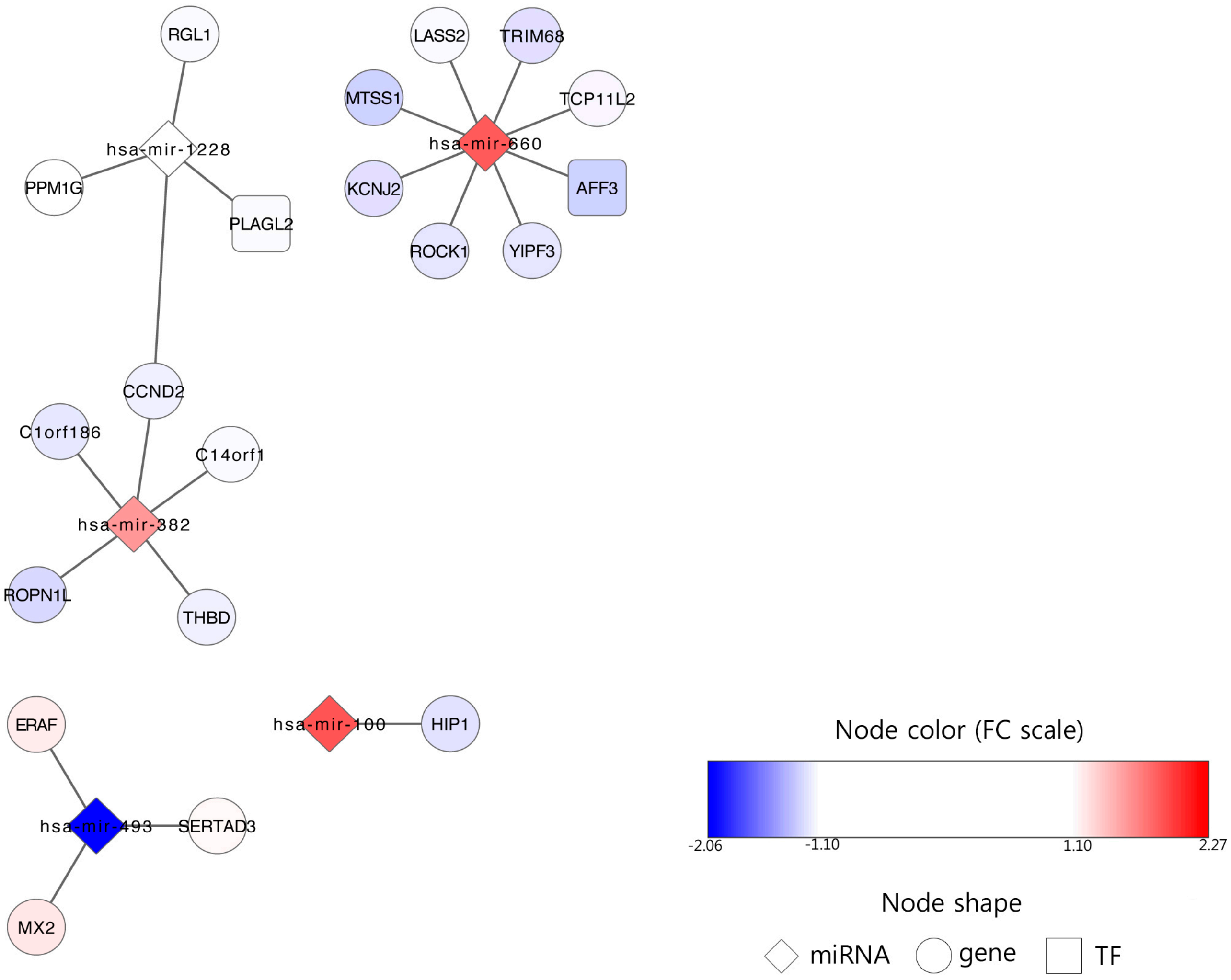

Supplementary table 1. The list of sncRNA which show a differential expression between relapse and remission phases in each group are shown, after applying the Rank Product algorithm. The fold-change (FC), p-value and false discovery rate values are shown.

RELAPSE VS REMISSION							
All				***Females***			
**sncRNA**	**FC**	**p-value**	**FDR**	**sncRNA**	**FC**	**p-value**	**FDR**
ACA40	2.53	8.98E-08	0.000	ACA40	9.21	0.00E+00	0.000
hsa-miR-1246	2.14	6.11E-06	0.003	hsa-miR-1246	6.83	0.00E+00	0.000
hsa-miR-1270	2.06	3.14E-05	0.007	hsa-miR-210	6.55	0.00E+00	0.000
ACA18	1.99	2.06E-04	0.018	hsa-miR-1270	5.08	0.00E+00	0.000
ACA24	1.91	6.86E-04	0.038	hsa-miR-183	4.96	8.98E-06	0.002
U70	1.83	1.87E-04	0.019	hsa-miR-18b	4.49	9.88E-05	0.016
hsa-miR-19a	1.77	2.21E-04	0.015	hsa-miR-98	3.70	1.17E-04	0.016
hsa-miR-335	1.77	2.78E-04	0.018	hsa-miR-331-5p	3.53	2.79E-04	0.028
hsa-miR-210	1.77	8.98E-06	0.003	hsa-miR-30e	3.51	4.58E-04	0.036
hsa-miR-18b	1.76	3.15E-05	0.006	hsa-miR-26b	3.46	1.80E-04	0.022
hsa-miR-18a	1.68	5.54E-04	0.034	U70	3.31	2.34E-04	0.026
hsa-miR-326	1.67	1.52E-04	0.017	hsa-miR-1224-5p	3.10	2.96E-04	0.025
hsa-miR-1224-5p	1.67	2.16E-04	0.017	ACA15	3.06	2.88E-04	0.027
hsa-miR-199a-3p	1.64	1.50E-04	0.019	hsa-miR-933	2.87	5.66E-04	0.042
hsa-miR-183	1.51	2.00E-04	0.019	hsa-miR-326	2.84	6.92E-04	0.048
hsa-miR-30e	1.49	1.62E-05	0.005	hsa-miR-1825	-1.63	2.43E-04	0.034
hsa-miR-17-star	1.45	6.73E-04	0.039	hsa-miR-504	-2.01	6.92E-04	0.041
hsa-miR-24-2-star	1.35	2.19E-04	0.016	hsa-miR-1280	-2.12	6.92E-04	0.041
hsa-miR-1202	1.33	7.63E-05	0.011	U81	-2.26	2.61E-04	0.029
hsa-miR-1825	-1.25	5.72E-05	0.016	SNORD4B	-2.31	8.63E-04	0.046
hsa-miR-671-3p	-1.72	1.35E-06	0.001	SNORD68	-2.33	9.70E-04	0.047
hsa-miR-455-3p	-1.77	5.12E-06	0.002	U57	-2.34	5.66E-04	0.042
hsa-let-7d-star	-1.84	3.59E-07	0.000	U78	-2.35	5.39E-04	0.043
				SNORD116-6	-2.36	5.66E-04	0.042
				hsa-miR-663	-2.38	9.16E-04	0.046
				U76	-2.40	5.39E-04	0.043
				U55	-2.41	8.36E-04	0.047
				hsa-miR-197	-2.50	5.21E-04	0.048
				hsa-miR-125a-5p	-2.52	6.47E-04	0.042
				hsa-miR-935	-2.54	3.32E-04	0.034
				U25	-2.55	2.34E-04	0.043
				U44	-2.56	2.43E-04	0.034
				hsa-miR-193a-5p	-2.71	2.43E-04	0.034
				hsa-miR-337-3p	-2.74	1.80E-04	0.040
				hsa-miR-766	-2.88	9.88E-05	0.028
				hsa-miR-455-3p	-3.13	8.98E-06	0.003
				hsa-let-7d-star	-3.73	0.00E+00	0.000
				hsa-miR-671-3p	-4.48	0.00E+00	0.000

Supplementary table 2. The list of sncRNA which show a differential expression between remission and healthy controls in each group are shown, after applying the Rank Product algorithm. The folc-change (FC), p-value and false discovery rate values are shown.

REMISSION VS CONTROLS											
All				***Females***				***Males***			
**sncRNA**	**FC**	**p-value**	**FDR**	**sncRNA**	**FC**	**p-value**	**FDR**	**sncRNA**	**FC**	**p-value**	**FDR**
hsa-miR-1228	1.61	3.26E-05	0.009	SNORD116-26	-1.40	9.69E-04	0.026	hsa-miR-1228	2.27	1.41E-04	0.031
hsa-miR-940	1.57	9.61E-06	0.004	hsa-miR-198	-1.46	4.31E-04	0.017	hsa-miR-100	1.87	2.57E-05	0.014
hsa-miR-197	1.41	1.97E-04	0.024	hsa-miR-210	-1.54	2.27E-04	0.011	hsa-miR-660	1.85	5.51E-05	0.020
hsa-miR-1825	1.34	1.51E-04	0.021	ACA20	-1.57	3.46E-04	0.015	hsa-miR-382	1.57	9.36E-05	0.026
hsa-miR-660	1.32	2.16E-06	0.001	hsa-miR-183	-1.58	2.53E-04	0.012	SNORD116-26	-1.57	5.04E-05	0.028
hsa-miR-200b-star	1.24	1.45E-04	0.023	hsa-miR-21	-1.59	2.85E-04	0.013	hsa-miR-493	-2.06	3.67E-05	0.041
hsa-miR-671-3p	1.17	4.54E-05	0.010	hsa-miR-148a	-1.60	5.85E-04	0.019	ACA40	-2.09	9.31E-05	0.035
hsa-miR-100	1.06	1.08E-04	0.020	hsa-miR-195	-1.63	7.43E-04	0.022				
hsa-miR-1246	1.01	2.18E-04	0.024	hsa-miR-30e	-1.66	9.90E-04	0.026				
hsa-miR-548a-3p	-1.04	4.08E-04	0.014	hsa-miR-485-5p	-1.66	5.33E-04	0.019				
hsa-miR-551b-star	-1.08	2.39E-04	0.024	hsa-miR-18b	-1.67	5.38E-04	0.018				
hsa-miR-1263	-1.10	5.70E-04	0.018	hsa-miR-886-5p	-1.68	1.54E-04	0.009				
hsa-miR-198	-1.13	5.09E-04	0.016	hsa-miR-1271	-1.69	4.33E-04	0.017				
hsa-miR-628-5p	-1.25	1.81E-04	0.008	U18A	-1.71	9.36E-04	0.026				
hsa-miR-183	-1.26	6.29E-04	0.018	hsa-miR-493	-1.73	5.13E-04	0.019				
hsa-miR-21	-1.28	1.20E-04	0.005	hsa-miR-199b-3p	-1.81	7.27E-04	0.022				
SNORD116-6	-1.29	4.12E-04	0.013	hsa-miR-26b	-1.86	1.50E-04	0.010				
hsa-miR-210	-1.35	6.24E-04	0.018	hsa-miR-487a	-1.87	7.72E-04	0.023				
hsa-miR-18b	-1.36	5.92E-04	0.017	hsa-miR-505	-1.89	1.52E-04	0.009				
hsa-miR-146b-3p	-1.42	7.80E-04	0.020	hsa-miR-379	-1.91	2.83E-04	0.013				
hsa-miR-30e	-1.42	7.76E-04	0.021	hsa-miR-30a	-1.92	6.68E-04	0.021				
hsa-miR-335	-1.42	3.07E-04	0.011	hsa-miR-30e-star	-1.92	1.85E-04	0.010				
hsa-let-7f	-1.44	7.52E-04	0.020	hsa-miR-409-5p	-1.94	3.58E-04	0.015				
hsa-miR-886-5p	-1.46	1.06E-04	0.005	hsa-miR-146b-5p	-1.96	8.27E-04	0.024				
SNORD116-26	-1.47	2.70E-07	0.000	hsa-miR-140-5p	-1.98	6.02E-06	0.001				
hsa-miR-140-5p	-1.47	2.36E-05	0.002	hsa-miR-27b	-2.01	5.13E-04	0.018				
hsa-miR-431	-1.50	3.11E-05	0.002	hsa-miR-543	-2.04	1.04E-04	0.007				
hsa-miR-382	-1.51	3.32E-06	0.001	hsa-miR-199a-3p	-2.08	1.68E-04	0.009				
hsa-miR-370	-1.52	2.42E-04	0.010	hsa-miR-432	-2.11	1.33E-05	0.001				
hsa-miR-152	-1.54	2.15E-04	0.009	hsa-miR-494	-2.12	6.68E-05	0.005				
U18A	-1.54	8.46E-04	0.021	hsa-miR-134	-2.15	1.55E-05	0.001				
hsa-miR-543	-1.56	8.75E-04	0.022	hsa-miR-433	-2.18	1.40E-05	0.001				
hsa-miR-26b	-1.60	3.80E-05	0.002	hsa-miR-431	-2.20	1.27E-05	0.002				
hsa-miR-487b	-1.63	7.73E-06	0.001	ACA40	-2.25	8.98E-08	0.000				
U46	-1.65	1.05E-04	0.005	hsa-miR-370	-2.26	2.24E-05	0.002				
hsa-miR-199a-3p	-1.66	2.89E-04	0.011	hsa-miR-654-3p	-2.34	2.19E-05	0.002				
hsa-miR-432	-1.66	1.44E-05	0.001	hsa-miR-192	-2.41	8.54E-06	0.001				
hsa-miR-146b-5p	-1.66	5.89E-04	0.018	hsa-miR-409-3p	-2.59	1.26E-06	0.000				
hsa-miR-192	-1.68	3.85E-05	0.002	hsa-miR-181a-2-star	-2.68	7.19E-07	0.000				
hsa-miR-30e-star	-1.70	8.54E-06	0.001	hsa-miR-487b	-2.73	8.98E-08	0.000				
hsa-miR-409-3p	-1.71	3.59E-05	0.003	hsa-miR-382	-2.80	0.00E+00	0.000				
hsa-miR-1271	-1.73	8.83E-05	0.005	hsa-miR-127-3p	-2.91	8.98E-08	0.000				
hsa-miR-433	-1.74	8.00E-06	0.001								
hsa-miR-134	-1.75	1.20E-05	0.001								
ACA20	-1.77	5.12E-06	0.001								
hsa-miR-31	-1.83	3.59E-05	0.003								
hsa-miR-493	-1.86	0.00E+00	0.000								
hsa-miR-494	-1.87	4.38E-05	0.003								
hsa-miR-127-3p	-2.09	8.98E-08	0.000								
ACA40	-2.18	0.00E+00	0.000								
hsa-miR-181a-2-star	-2.34	0.00E+00	0.000								

Supplementary Table 3. Clinical and demographic data on patients and healthy controls included in the blood and cultured peripheral blood mononuclear cell miRNA microarray experiments.

	Blood samples		Cultured PBMCs	
	Patients (n=24)	Healthy Controls (n=24)	Patient (n=1)	Healthy Control (n=1)
Age, years (mean ± sd) (range)	40.21 ± 10.13 (23-66)	39.96 ± 10.67 (23-66)	40	29
Gender				
Females	14 (58%)	14 (58%)	1	1
Males	10 (42%)	10 (42%)	-	-
EDSS (mean ± sd) (range)	2.69 ± 1.94 (0-7.5)	-	2.5	-
AOO, years (mean ± sd) (range)	30.63 ± 10.73 (11-65)	-	21	-
Disease duration, years (mean ± sd) (range)	9.58 ± 8.05 (1-33)	-	19	-

Abbreviations. EDSS: expanded disability status scale; AOO: age of onset; sd: standard deviation.
